# Central nervous system aspergillosis misdiagnosed as Toxoplasma gondii encephalitis in a patient with AIDS: a case report

**DOI:** 10.1186/s12981-022-00468-x

**Published:** 2022-09-08

**Authors:** Hong-Hong Yang, Xue-Jiao He, Jing-Min Nie, Shao-Shan Guan, Yao-Kai Chen, Min Liu

**Affiliations:** 1 Division of Infectious Diseases, Chongqing Public Health Medical Center, 109 Baoyu Road, Shapingba District, Chongqing, 400036 China; 2 Hunan Sagene Medical Laboratory Limited, Changsha, 410036 Hunan China

**Keywords:** CNSAG, AIDS, TE, mNGS, tNGS

## Abstract

**Background:**

Patients with acquired immunodeficiency syndrome (AIDS) tend to suffer from several central nervous system (CNS) infections due to hypoimmunity. However, CNS aspergillosis (CNSAG) is extremely rare and difficult to diagnose. Thus, it is easily misdiagnosed.

**Case presentation:**

We reported a 47-year-old male AIDS patient with ghosting vision and anhidrosis on the left head and face. He was accordingly diagnosed with Toxoplasma gondii encephalitis (TE) at other hospitals, for which he received regular anti-Toxoplasma gondii and anti-human immunodeficiency virus (anti-HIV) treatment. Then, the patient was transferred to our hospital due to a lack of any improvement with the prescribed treatment. The patient's neurological examination revealed no abnormalities at admission, only a slight change in the cerebrospinal fluid. His cranial magnetic resonance imaging (MRI) revealed multiple abnormal signals in the brain parenchyma, and his blood was positive for Toxoplasma gondii IgG antibody. The initial diagnosis at our hospital was also TE. Considering the poor efficacy of anti-TE treatment, cerebrospinal fluid metagenomics next-generation sequencing (mNGS) was performed, but no pathogenic bacteria were detected. However, Aspergillus fumigatus was detected in the cerebrospinal fluid via targeted next-generation sequencing (tNGS) and bronchoalveolar alveolar lavage fluid via mNGS. The diagnosis was accordingly revised to CNSAG combined with his other clinical manifestations. After administering voriconazole antifungal therapy, the patient’s symptoms were relieved, with improved absorption of the intracranial lesions.

**Conclusions:**

The present case experience indicates the need for clinicians to strengthen their understanding of CNSAG. Moreover, for patients with diagnostic difficulties, early mNGS and tNGS (using biological samples with only a few pathogens) are helpful for early diagnosis and treatment, potentially allowing patients to achieve favorable outcomes.

## Introduction

Central nervous system aspergillosis (CNSAG) is a rare disease related to low immunity, The CNSAG incidence rate is high in patients with solid organ transplantation or hematological diseases as well as in patients undergoing long-term immunosuppressive treatment [1]. Due to hypoimmunity, patients with acquired immunodeficiency syndrome (AIDS) also experience various central nervous system (CNS) infections, such as tuberculous meningitis, Toxoplasma gondii encephalitis (TE), and cryptococcal meningitis. However, CNSAG in the context has rarely been reported [2, 3]. The clinical symptoms of CNSAG are nonspecific and associated with the degree of intracranial involvement, the condition often manifests as headache, focal neurological dysfunction, altered mental state, or vasculitis [4, 5]. Routine examination of the cerebrospinal fluid, biochemical detection, and cranial imaging examination are also limited due to low specificity [6]. The diagnosis of CNSAG is mainly based on positive cerebrospinal fluid culture or biopsy to identify the pathogens; however, this process is time-consuming with a low positive rate, making early and accurate diagnosis difficult. At present, the wide application of metagenomics next-generation sequencing (mNGS) technology for extensive and unknown pathogens has dramatically improved the etiological detection rate. However, mNGS has some detection limitations when applied to cerebrospinal fluid with a low count of pathogenic bacteria. Targeted next-generation sequencing (tNGS) is a highly sensitive detection technology for known pathogens that can significantly improve the clinical detection rate of pathogens that are difficult to detect and have low copy infections. tNGS compensates for the deficiencies of mNGS and assists in early diagnosis and early treatment to improve the patient survival rate. We report herein the case of a patient with CNSAG that was misdiagnosed as TE.

## Case report

The patient is a 47-year-old, married, male construction worker. The patient was treated at a hospital in Chongqing on May 10, 2021 due to cough, shortness of breath, and other discomfort and was accordingly diagnosed with pneumocystis carinii pneumonia (PCP) and AIDS. His baseline CD4+ T-cell count and HIV-RNA level were unknown. After anti-PCP treatment (with compound sulfamethoxazole tablets combined with prednisone), his symptoms were relieved. Thereafter, antiretroviral therapy (ART), including lamivudine (3TC), tenofovir (TDF), and efavirenz (EFV) was initiated. He took the ART and compound sulfamethoxazole tablets regularly.

He was hospitalized on August 19, 2021, due to ghosting vision for > 2 months and anhidrosis on the left head and face for the past 20 days. Over 2 months before hospital admission, the patient experienced bilateral ghosting vision and limited rotation of the left eye without any obvious induction. Moreover, he had no fever, dizziness, headache, tinnitus, decreased hearing and vision, visual rotation, syncope, disturbance of consciousness, nausea, vomiting, incontinence, abdominal pain, diarrhea, or other discomforts. Accordingly, the patient was treated at a hospital in Chongqing. His cranial MRI revealed multiple intracranial abnormal enhancement signals with edema. Furthermore, routine examination and biochemical detection of the cerebrospinal fluid indicated minimal abnormalities. He was diagnosed with TE and AIDS. After treatment with azithromycin combined with compound sulfamethoxazole tablets against Toxoplasma gondii for 2 weeks, the patient reported slight improvement in his ghosting vision. Then, he was discharged without any imaging re-examination and was continued on the original anti-Toxoplasma gondii treatment after discharge. Then, 20 days before his readmission (the patient had been receiving continuous anti-Toxoplasma gondii treatment until his hospitalization in our hospital), the patient still had anhidrosis on the left head and face. In addition, his bilateral ghosting vision due to unknown causes had worsened, accompanied by limited rotation of the left eye, slight cough, and less sputum production. He had no tinnitus, decreased hearing and vision, visual rotation, blackness, epilepsy, disturbance of consciousness, or other discomforts. His brain MRI re-examination in other hospitals indicated that the intracranial lesions had increased significantly. The patient was subsequently transferred to our hospital for further treatment.

**Fig. 1:**
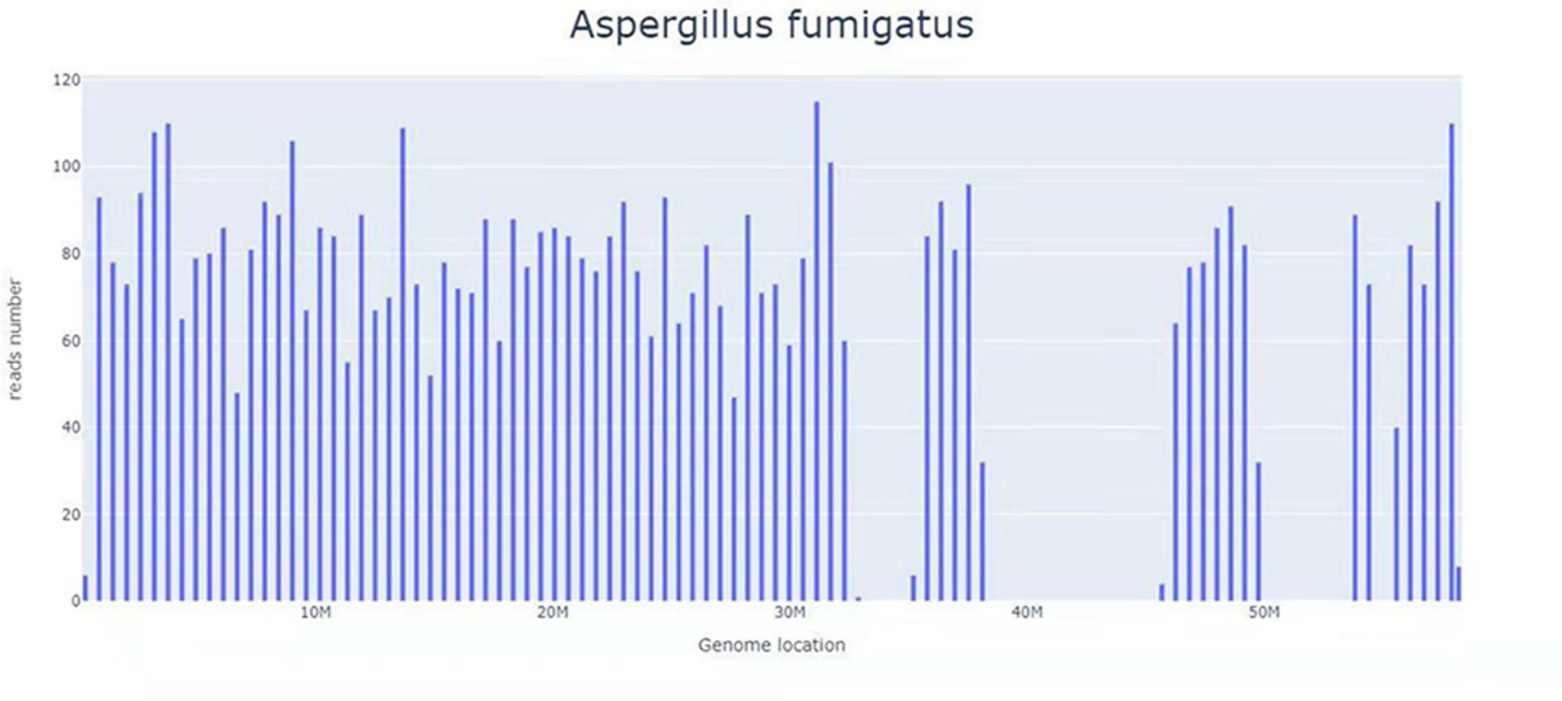
The mNGS test results for bronchofiberscope alveolar lavage fluid

The results of the patient’s physical examination on admission were as follows: normal vital signs and a clear mind, he answered to the point; the bilateral pupils were equal in size and circular-shaped, showing sensitivity to light reflection; the left eye had external rotation disorder, but the right eye had normal activity; his lungs had clear respiratory sound, without rhonchus and moist rales; no abnormalities were detected upon cardiac and abdominal physical examination; the muscle strength and tension of the limbs were normal; pain and temperature sensation were normal, and bilateral pathological signs were negative. The results of his laboratory examination were as follows: cranial MRI showed patchy mixed long T1 and T2 signals in the bilateral frontal lobes and mixed high signals in flair, surrounded by massive edema. Enhanced scanning revealed that multiple intracranial lesions were circular, nodal, and enhanced, and the intracranial pia mater was slightly thickened. His chest CT indicated that the lungs were scattered with patchy, strip-shaped, nodule-like, acinar nodular increased shadows with uneven density and some unclear boundaries. His routine blood test results were as follows: white blood cells 3.08 × 10^9^/L, neutrophils 2.29 × 10^9^/L, and cerebrospinal fluid: pressure 140 mmHg, routine examination of the cerebrospinal fluid revealed a positive result for Pandy’s test. His cerebrospinal fluid biochemistry was as follows: total protein 498.77 mg/L, microalbumin 360.7 mg/L, β2 microglobulin 5.26 mg/L, glucose 3.41 mmol/L, and chloride 128.8 mmol/L. The following test results were obtained: CD4^+^ T-cell count 57 cells/µL, CD8^+^ T-cell count 322 cells/µL, CD4/CD8: 0.18; Toxoplasma gondii IgG antibody in the blood: positive, Toxoplasma gondii IgM antibody and DNA: negative, galactomannan (GM) test in the blood, (1,3)-β-d glucan (BG), *Talaromyces* marneffei antigen, Cryptococcus neoformans antigen, latent tuberculosis infection, and cytomegalovirus DNA: negative. Cerebrospinal fluid staining with India ink, cryptococcal antigen, cytomegalovirus DNA, tuberculosis fluorescence staining, tuberculosis sandwich cup, bacterial and fungal cultures: were all negative. The primary diagnosis was: (1) TE(?), and (2) AIDS. The patient continued to receive 3TC/TDF/EFV anti-human immunodeficiency virus (anti-HIV) treatment and azithromycin combined with compound sulfamethoxazole against Toxoplasma gondii. After 1 week of treatment, the symptoms of ghosting vision and facial anhidrosis did not improve. Per a past report, patients with AIDS complicated with TE have shown significant curative effects after regular treatment for 2–4 weeks [7]. Therefore, we wondered whether TE was erroneously diagnosed. For precise diagnoses, the cerebrospinal fluid was collected for mNGS examination (RDP-seq^®^, Guangzhou Sagene Biotechnology Co., Ltd.), and the results indicated no possible pathogenic bacteria, such as Toxoplasma gondii, CMV, and Cryptococcus. Then, tNGS examination was performed on the cerebrospinal fluid sampled previously (Guangzhou Sagene Biotechnology Co., Ltd.). The results indicated Aspergillus fumigatus (number of sequences: 1). CNSAG was not diagnosed immediately as the number of t-NGS sequences in the cerebrospinal fluid was extremely low. Considering that Aspergillus fumigatus usually invades the lungs and that lesions were present in the patient’s lungs, fiberoptic bronchoscopy was immediately performed. The bronchoalveolar lavage fluid (BALF) was collected for mNGS examination (RDP-seq^®^, Guangzhou Sagene Biotechnology Co., Ltd.), and the results showed a large number of Aspergillus fumigatus (number of sequences: 5842, the degree of confidence: 99%) (Fig. 1).

**Fig. 2:**
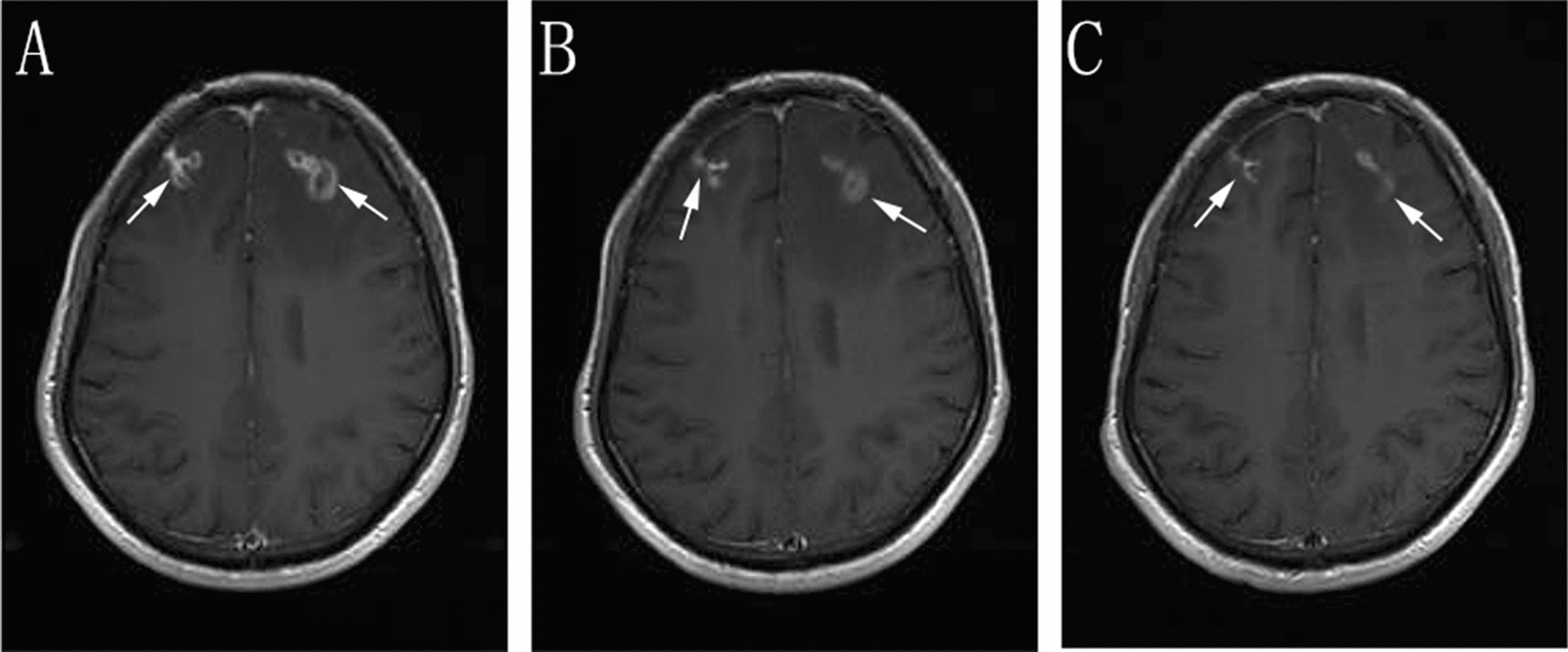
Enhanced scanning of cranial MRI **A** before treatment: multiple intracranial circular and small-nodular enhanced lesions, as indicated by the arrow, **B** two weeks after treatment **C** six weeks after treatment, **B** and **C** indicate that the intracranial lesions were absorbed and improved compared with those before treatment

**Fig. 3:**
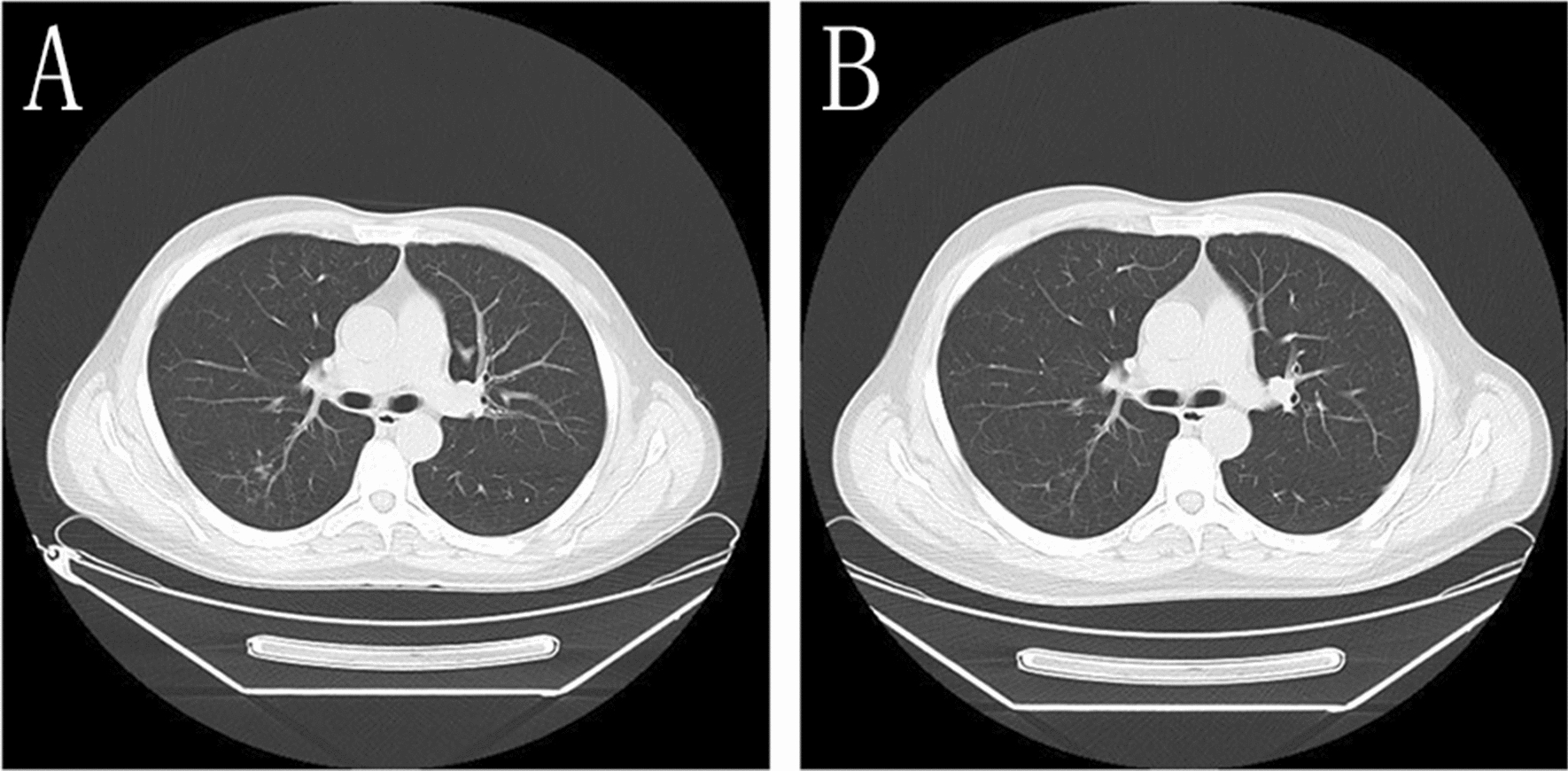
Chest CT **A** Before treatment, the lung tissues seemed scattered with patchy, strip-shaped shadows, nodule-like and increased acinar nodular shadows with uneven density and some unclear boundaries were noted. **B** After 6 weeks of treatment, chest CT showed that the lesions were resolved

In addition, the BALF-GM test result was positive. Based on the patient’s symptoms, signs, laboratory tests, and past treatment reactions, the diagnosis was corrected to CNSAG, invasive pulmonary aspergillosis (IPA), and AIDS. Voriconazole (intravenous drips of 6 mg/kg/day q12-h on the first day, followed by intravenous drip of 4 mg/kg/day q12-h) was administered for antifungal treatment. Due to the interaction between voriconazole and EFV, the antiviral treatment regimen was revised to 3TC, TDF, and dolutegravir (DTG). After 2 weeks of this treatment, the patient’s ghosting vision and facial anhidrosis were relieved. The routine examination of cerebrospinal fluid revealed normal biochemistry. His cranial MRI indicated that the lesions in the frontal lobe were absorbed, and the surrounding edema was alleviated (Fig. 2). The patient was discharged and continued on oral voriconazole treatment (200 mg/kg/day). After 6 weeks of antifungal treatment, the patient's ghosting vision and facial anhidrosis were significantly relieved. Re-examination of his cranial MRI indicated that the lesions in the frontal lobe were significantly absorbed and improved, and the surrounding edema was alleviated (Fig. 2). His chest CT showed focal absorption (Fig. 3).

## Discussion

Invasive aspergillosis (IA) is a common severe infection in immunosuppressive patients, and high mortality is noted when IA is not treated in a timely fashion. Aspergillus fumigatus is the most common pathogenic bacterium in IA [4]. IA commonly invades the lungs in AIDS patients, but it can spread to other organs, including the CNS, albeit less commonly. When Aspergillus invades the CNS, the positive culture rate is low due to the low count of pathogenic bacteria in the cerebrospinal fluid [8]. Moreover, brain tissue biopsy is not a universal approach and is inaccessible at several medical institutions, which often leads to the misdiagnosis of CNSAG, thereby affecting the optimal anti-infection treatment approach.

The present patient had a history of AIDS, with a low level of CD4 T-lymphocytes. Neurological symptoms appeared earlier, and relatively minimal cerebrospinal fluid changes were noted. However, his brain MRI revealed multiple focal lesions in the brain parenchyma, surrounded by edema, whereas his enhanced MRI revealed circular enhancement, similar to the TE imaging findings reported elsewhere [9]. The patient’s blood was positive for Toxoplasma gondii IgG, and TE is one of the most common CNS opportunistic infections found in AIDS patients [10]. The present findings may be crucial to the misdiagnosis of this case as TE by other hospitals and even our hospital. However, after regular treatment for 2–4 weeks, the clinical symptoms and intracranial imaging results of most AIDS patients with TE show good curative effects on [7]. A relatively poor effect was noted in this patient despite regular anti-Toxoplasma gondii treatment for > 4 weeks. Furthermore, this patient had received regular sulfamethoxazole tablets after being released from the hospital three months earlier due to PCP, and research shows that Toxoplasma gondii encephalopathy rarely occurs under preventative treatment with sulfonamides [11]. In this condition, we must keep in mind whether the diagnosis is correct, and need to take a further step to determine the true etiology and pathogenesis, such as other pathogen infections or cancers.

mNGS is a novel gene detection technology that can theoretically detect all pathogens in samples rapidly without any bias. This technology targets bacteria, viruses, fungi, and parasites, making it especially suitable for rare, emerging, and atypical pathogens [12–14]. Studies have shown that mNGS is more sensitive than traditional culture methods in clinical conditions such as blood stream, CSF and respiratory infections [15, 16]. However, no pathogenic bacteria were detected in the patient’s CSF. Similarly, Miller [17] reported that *Aspergillus fumigatus* in CSF was likely missed by mNGS testing because given the high background noted in samples and weakly positive results. tNGS is a high-throughput sequencing detection technology that is used to identify specific known pathogens. It has been reported that tNGS has a number of advantages over mNGS, including its sensitivity, timeliness, and cost. In addition, tNGS is especially suitable for detecting common, infectious pathogens in a clinical setting that are difficult to detect and present at low copy numbers [18, 19]. The results of tNGS in the CSF revealed Aspergillus fumigatus (number of sequences: 1). Due to the low number of t-NGS sequences in the cerebrospinal fluid, CNSAG was not diagnosed immediately. Nonetheless, IA seemed to be a probable diagnosis.

Past studies have shown that IA occurs in patients with various risk factors, including HIV infection and steroid therapy [20]. A recently documented risk factor for IA is exposure to environmental *Aspergillus* spores at construction sites. A patient who had been working as a laborer at a construction site was a victim of Aspergillus infection due to the inhalation of spores released at the construction site [21]. This patient had the above risk factors that may have predisposed to IA. The patient also had respiratory symptoms, such as cough and expectoration, with multiple morphologically distinct lesions noted on chest CT. At this point, we needed a different approach, so BALF was collected for mNGS detection, and the results revealed the presence of numerous of Aspergillus fumigatus. The results of the BALF-GM test were also positive, indicating IA infection. The patient was accordingly diagnosed with CNSAG and IPA. Early diagnosis of CNSAG is essential for successful treatment. Therefore, for the diagnosis of CNS infection in HIV patients, if a pathogen responsible for infection cannot be identified through normal molecular testing, mNGS should be performed as early as possible. For pathogens that are difficult to identify, such as *Aspergillus fumigatus*, *Mycobacterium tuberculosis*, cryptococcal meningitis, or low-copy pathogens, tNGS may represent a good tool. In this case, the patient was at high risk of invasive fungal and tuberculosis infection. Considering the low detection rate of pathogens associated with these two types of infection, tNGS was conducted. In addition, as an auxiliary technology for clinical pathogen diagnoses, the detection results of tNGS are affected by several factors, including sample quality, pathogen content, detection technology, and method of analysis. Thus, the number of sequences cannot be used as the standard for clinical diagnosis. In the future, we need to determine the diagnostic basis through comprehensive analysis combined with deep exploration in future clinical practice.

Voriconazole is the first-line treatment for IA [22], and it crosses through the blood‒brain barrier. Therefore, the patient was administered voriconazole antifungal treatment. After 6 weeks of this treatment, the patient's ghosting vision and facial anhidrosis symptoms were completely relieved. Reexamination of the cranial MRI and chest CT showed that the lesions were absorbed and had improved. Given the rarity of this disease, early diagnosis of IA is extremely difficult. This noted is also demonstrated by the large number of cases that are diagnosed postmortem. Fortunately, this patient was an exception to this rule and was diagnosed in a timely manner and successfully treated. Notably, several core drugs used in the antiviral treatment of AIDS are metabolized by liver cytochrome P450 isozyme. These drugs can subsequently have a serious interaction with voriconazole, which is metabolized by the same metabolic pathway. Hence, clinicians should remain vigilant when formulating antiviral treatment regimens to avoid altering the efficacy of antiHIV and anti-fungal therapy.

## Conclusion

First, it is worth noting that up to 7.9% of the healthy population also harbors Toxoplasma gondii IgG [23]. This positive result only indicates that a patient was previously infected with Toxoplasma gondii, and it cannot be used as a diagnostic index of TE. Second, the clinical manifestations, routine examination of the CSF, and biochemical detection of CNSAG lack specificity, and some of its imaging manifestations are similar to those of TE. Therefore, one should remain vigilant to avoid such a serious misdiagnosis. Third, for HIV patients with possible intracranial infection, mNGS should be performed as early as possible if a pathogen cannot be identified by normal molecular testing, and tNGS can be recommended to improve the detection rate for *Aspergillus fumigatus* and tuberculosis infections with low sensitivity. Finally, the search for pathogens of intracranial infection should not be limited to intracranial locations. It is necessary to assess multiple sites as part of a comprehensive analysis.

## Data Availability

The data that support the findings of this study are openly available.

## Abbreviations

AIDS: Acquired immunodeficiency syndrome
CNS: Central nervous system
CNSAG: Central nervous system aspergillosis
CSF: Cerebrospinal fluid
HIV: Human immunodeficiency virus
TE: Toxoplasma gondii encephalitis
mNGS: Metagenomics next-generation sequencing
TE: Toxoplasma gondii encephalitis
tNGS: Targeted next-generation sequencing
IPA: Invasive pulmonary aspergillosis
PCP: Pneumocystis carinii pneumonia
ART: Antiretroviral therapy
GM: Galactomannan
BG: (1,3)-β-d-glucan
